# Single Dose of the Attention Training Technique Increases Resting Alpha and Beta-Oscillations in Frontoparietal Brain Networks: A Randomized Controlled Comparison

**DOI:** 10.3389/fpsyg.2018.01768

**Published:** 2018-09-20

**Authors:** Mark M. Knowles, Adrian Wells

**Affiliations:** ^1^Manchester University Hospitals NHS Foundation Trust, Manchester, United Kingdom; ^2^Division of Psychology and Mental Health, The University of Manchester, Manchester, United Kingdom; ^3^Greater Manchester Mental Health NHS Foundation Trust, Manchester, United Kingdom

**Keywords:** attention, attention training technique, therapeutics, psychophysiology, electroencephalography, executive control, metacognitive therapy

## Abstract

The Attention Training Technique (ATT) was developed with the aim of reducing self-focused attention and increasing executive control as part of metacognitive therapy. So far there is a paucity of data on the neurophysiological effects of ATT. In the present study we tested for specific effects to determine if attention control components of ATT elicit a specific signature that is different from passive listening. Thirty-six healthy volunteers were randomized to an active (follow instructions) or control (ignore instructions) condition. Resting state EEG was recorded for 3 min with eyes open and eyes closed before and after exposure to training, and the power of the theta, alpha, and beta-bands were analyzed in frontal, midline, and posterior electrodes. The active ATT condition enhanced alpha and beta-band activity during eyes-open, and frontal alpha during eyes-closed (*p* < 0.005). Frontoparietal changes in Alpha were generally accompanied by changes in Beta in the same brain regions of interest. However, these associations were largely significant in the active ATT rather than the control condition. No between-group differences were observed in the Theta-band. These results suggest a single dose of attention training increases alpha and beta-oscillations in frontoparietal networks. These networks are associated with top-down attentional or executive control.

## Introduction

The Attention Training Technique (ATT; Wells, 1990) is a metacognitive treatment strategy grounded in the Self-Regulatory Executive Function Model (S-REF; Wells and Matthews, 1994, 1996) of psychological disorder. According to S-REF theory, a specific pattern of thinking called the ‘Cognitive Attentional Syndrome’ (CAS) is assumed responsible for the maintenance of emotional distress. The CAS consists of unhelpful modes of processing including inflexible self-focused attention, threat-orientated attention biases, and worry and rumination. When activated, the CAS leads to a loss of cognitive resources and locks individuals into extended patterns of negative processing of threat. As a result, psychological change is impeded because the processing resources required for efficient top-down self-regulation are reduced. A key feature of metacognitive therapy (MCT; Wells, 2000, 2009) is the explicit modification of maladaptive attentional strategies and the knowledge concerning them. ATT was developed as part of MCT to help moderate CAS activation by increasing top-down attentional control and flexibility. ATT consists of auditory attentional exercises that require individuals to engage in executive control skills including selective attention, divided attention, and attention switching (for a comprehensive overview of ATT, see Wells, 2009).

A large body of experimental and clinical data supports the contention that components associated with the CAS are linked to negative emotional outcomes. Studies include those examining worry and rumination (e.g., Nolen-Hoeksema, 1991; Capobianco et al., 2018), inflexible attention (e.g., Liu et al., 2002; Kolur et al., 2006), attentional biases (e.g., Mogg and Bradley, 2005; Bar-Haim et al., 2007), and inefficient cognitive control (e.g., Arnsten and Rubia, 2012; Wiers et al., 2013). Aside from supporting the S-REF model, such studies also complement wider views within neuroscience highlighting the critical role that executive control processes play within psychopathology. For example, decreased prefrontal function has been observed across multiple psychiatric conditions and is thought to reflect inefficiency in top-down regulatory processes including attentional flexibility, working memory, response inhibition, and the planning and execution of adaptive responses (Miller, 2000; Miyake et al., 2000; Porter et al., 2007). An implication of these results is that treatments integrating strategies specifically designed to attenuate deficits in executive control are more likely to prove efficacious (Siegle et al., 2007). In particular, treatments such as ATT which aim to increase aspects of attentional control and flexibility are predicted to yield improved functional and neurocognitive outcomes (Wells, 1990; Wells and Matthews, 1996; Siegle, 1999; Ottowitz et al., 2002).

Although originally developed as part of MCT, ATT has since been recognized as an effective stand-alone treatment for both anxiety and depressive disorders (e.g., Fergus and Bardeen, 2016; Knowles et al., 2016). Furthermore, a number of efficacy trials have demonstrated that the specific attentional processes targeted by the technique (e.g., inflexible self-focused attention, attentional bias) are associated with improved executive control and symptom relief (e.g., Sharpe et al., 2010; Callinan et al., 2014; Fergus et al., 2014; Nassif and Wells, 2014). In addition to this clinical and experimental data, a small number of studies are also beginning to uncover the neurophysiological effects of the technique. For example, initial neuropsychological and functional magnetic resonance imaging (fMRI) data suggests that Cognitive Control Training, which combines ATT with a working memory task, enhances activity within the dorsolateral prefrontal cortex (dlPFC), improves executive control, and disrupts amygdala activity in unipolar depression (Siegle et al., 2007, 2014). Furthermore, initial data from functional near-infrared spectroscopy (fNIRS) studies has also demonstrated increased blood oxygenation in the right inferior frontal gyrus, the right dorsolateral prefrontal cortex, and the superior parietal lobule during ATT in comparison to a control condition (Rosenbaum et al., 2018).

In the present study, we sought to provide further insight regarding the neurophysiological effects of ATT by using electroencephalography (EEG) to evaluate change in oscillatory activity across the scalp. EEG methodology was selected for two primary reasons: first, we were interested to see whether change in tonic frequency power following exposure to ATT would yield increased activity in known areas associated with top-down executive control. For example, it is well established that alpha and beta oscillations are generated by frontoparietal executive control networks (e.g., Capotosto et al., 2009; Sauseng et al., 2009; Thut et al., 2011) and are thought to reflect engagement of executive skills including attentional control and the regulation of working memory (e.g., Hanslmayr et al., 2007; Klimesch et al., 2007; Haegens et al., 2011; Handel et al., 2011). It was therefore hypothesized that engagement of ATT would yield increased changes in alpha and beta-band activity in frontoparietal regions. Second, we were interested to learn whether the effects of ATT would yield a different oscillatory signature to other known forms of attention modification. For example, although increased theta activity has been traditionally linked to short and long-term memory (e.g., Fell et al., 2003; Vertes, 2005), it has also been reliably observed to reflect a relaxed, drowsy state during mindfulness and meditation-based techniques (for reviews, see Cahn and Polich, 2006; Ivanovski and Malhi, 2007; Chiesa and Serretti, 2010; Travis and Shear, 2010). It was therefore hypothesized that in comparison to these findings, the effects of ATT would yield little or no change in theta-band activity.

In order to test our predictions, we designed a randomized controlled comparison in which participants were assigned to either an active (follow ATT instructions) or control (listen passively but do not follow ATT instructions) condition. Resting-state EEG data were recorded before and after exposure to the ATT and tonic power change was investigated in the three frequency bands of interest: alpha, beta, and theta. This allowed us to separate the presumed mechanistic effects of ATT (engaging in attentional control strategies) from simple exposure to a therapeutic listening task. Hence, in doing so, this design provided us with a structurally equivalent control condition that allowed EEG within and between-group changes to be attributed to manipulation of the IV (engaged vs. passive exposure to ATT). Furthermore, as this was one of the first known EEG studies to evaluate the effects of ATT, we recruited a non-clinical group of healthy subjects whom were naïve to the technique. Thus, participants were not socialized to the metacognitive model as would normally be expected in routine clinical practice. This helped us protect against possible measurement bias and placebo effects, and also prevented the investigated mechanism (engagement of ATT’s attentional exercises) from being disturbed by the influence of medication and/or psychopathology. From an ethical point of view, it is also important to first establish non-clinical neurophysiological effects which future clinical samples can be compared against (thus avoiding unnecessary testing of the latter group).

## Materials and Methods

This study was conducted in accordance with the Declaration of Helsinki (World Medical Association, 1964) and was approved by the University of Manchester Ethics Committee (ref number: 13214).

### Participants

Thirty-six student volunteers (22 female, 24.33 ± 6.99) gave written informed consent to take part in the study. Participants were recruited from the University of Manchester via poster advertisement and received either course credits or monetary remuneration for taking part. All participants had normal or corrected vision, were right-handed, and had no current or historical neurological or psychiatric conditions.

Participants completed a number of validated self-report measures prior to the trial to ensure equivalence between independent groups on measures of attentional control, metacognition, and current mood: the Attentional Control Scale (ACS: Derryberry and Reed, 2002), the Metacognitions Questionnaire-30 (MCQ-30: Wells and Cartwright-Hatton, 2004) and the UWIST Mood Adjective Checklist (UMACL: Matthews et al., 1990). The UWIST was also measured post-ATT in order to establish whether any change in mood occurred as a result of the technique (see Results). Participants also completed a post-manipulation check immediately after the study. This measure consisted of two questions: (1) ‘How much did you find yourself moving your attention around as instructed during the audio recording?’ and (2) ‘How much did you find yourself listening passively without moving your attention around during the audio recording?’ Participants were required to record their responses on a 0–100% Visual Analogue Scale (VAS).

### Experimental Procedure

Participants were randomly assigned^1^ to an Active Condition (AC; *n* = 18, 10 female) or a Control Condition (CC; *n* = 18, 12 female) and all listened to the ATT recording. Those in the AC were required to follow ATT instructions (participant instructions: ‘Please listen to the audio recording. You are required to follow the instructions’) and those in the CC were required to ignore the instructions (participant instructions: ‘Please listen to the audio recording. You are required to listen passively without following the instructions’). Participants were required to complete the post-manipulation check and a measure of current mood (UWIST) following ATT. The duration of the experiment was approximately 24 min: pre-resting state (6 min), ATT (12 min), post-resting state (6 min). Participants were debriefed following the study. There were no differences between groups on any of the pre-trial measures.

### EEG Recording

Continuous EEG was recorded at rest before and after exposure to ATT. Each recording lasted approximately 6 min in duration, with 3 min eyes-open (EO), and 3 min eyes-closed (EC). The order of EO and EC was randomly assigned and then counterbalanced across participants. The experiment was conducted in a light- and sound-attenuated, electrical shielded room at ambient temperature. Participants were seated comfortably on a chair and were requested to minimize eye-blinks and physical movements during recording. Participants were monitored during recording to ensure they did not fall asleep. EEG data were recorded using a 64-electrode BioSemi ActiveTwo amplifier conforming to the international 10–20 system (Jasper, 1958). Electrodes were attached in standard formation (details of BioSemi referencing and grounding conventions^2^). The signal was digitized at 512 Hz with an open passband from 0.01 to 100 Hz. Horizontal and vertical electro-oculograms were recorded using separate electrodes placed above and below the right eye and at the outer canthi of both eyes.

### Spectral Analysis

Continuous EEG data were imported into BrainVision Analyser (Brain Products GmbH, 2015). Data were re-referenced to the common average of electrodes across the scalp. Independent Components Analysis (ICA) was used across all for recordings (12 min in total) to remove ocular artifacts. Data were then reconstructed and segmented into 1s epochs, and spectral analysis was conducted using Fast-Fourier transformation (FFT) within pre-defined bands: Theta (4–7 Hz), Alpha (8–12 Hz), Beta (13–30 Hz). This yielded FFT average power values for each EEG frequency band expressed in log units, 10^∗^log_10_(μV^2^/Hz), as a measure of frequency density (activity) in all four recordings (pre-resting state EO/EC, post-resting state EO/EC).

Three topographic regions of interest (ROIs) were calculated by averaging power values across the following electrode sites: Anterior (AF7, Fp1, Fpz, Fp2, AF8, AF3, AFz, AF4, F7, F5, F3, F1, Fz, F2, F4, F6, and F8), Midline (FT7, FC5, FC3, FC1, FCz, FC2, FC4, FC6, FT8, T7, C5, C3, C1, Cz, C2, C4, C6, T8, TP7, CP5, CP3, CP1, CPz, CP2, CP4, CP6, and TP8), Posterior (P7, P5, P3, P1, Pz, P2, P4, P6, P8, PO7, PO3, POz, PO4, PO8, O1, Oz, and O2). Prior to statistical analysis, all data were normalized using natural logarithm (In) transformation and then pre-to-post resting-state change indices were calculated for each condition (i.e., post-minus pre-baseline resting state values). These represented unitary values of tonic power change following exposure to ATT and were assumed to reflect the extent to which neuronal synchrony was increased or decreased. The use of unitary index values was also selected in order to reduce the error variance for statistical analysis.

## Results

All analyses were performed using IBM SPSS v22 (IBM Corp, 2013). The initial phase of analysis evaluated whether any differences were observed between or within groups on the pre-selected measure of mood state (UWIST), and whether any between-group differences were observed on the post-manipulation check (which was designed to assess compliance with the task instructions). This was followed by a planned evaluation of differences between groups on EEG tonic power changes across the frequency bands (alpha, beta, and theta). Here, the primary variable of interest in the EEG data was the effect of engagement with ATT (active condition) on spectral power in comparison to non-engagement/passive listening of ATT (control condition). Finally, an unplanned exploration of the correlation coefficients between band-power changes across both conditions was also conducted in order to learn more about whether ATT yielded a different oscillatory signature in comparison to that reported for other forms of attention modification (such as mindfulness and meditation). Given the pilot nature of these data, no specific corrections were employed for multiple comparisons during phase 2 and 3 of the analysis: this decision was taken to reduce the possibility of Type 2 errors given the relatively small sample size obtained. While we recognize that this limits the reliability of our findings, we felt that this was the most appropriate action to take given that the use of corrections may have obscured any possible effects.

Phase 1: In order to examine pre-to-post change in mood state, a 2 (condition) × 2 (time) mixed analysis of variance (ANOVA) was conducted on the four subscales comprising the UWIST – Tense Arousal (TA), Energetic Arousal (EA), Hedonic Tone (HT), and Anger Items (AI) – where condition was a between-subjects factor, and time was a within-subjects factor. No significant main or interaction effects were observed on any of the subscales (all *p* > 0.05) indicating that mood state did not differ between time points. In order to assess whether participants followed experimental instructions, one-way ANOVAs were conducted on the post-manipulation checks. A significant difference was observed between groups on Question 1 [*F*(1,35) = 300.32, *p* < 0.001], with those in the AC (83.33 ± 11.11) yielding higher scores than those in the CC (19.83 ± 10.87). In contrast, a significant difference was observed between groups on Question 2 [*F*(1,35) = 141.99, *p* < 0.001], with those in the AC (19.17 ± 11.66) yielding lower scores than those in the CC (77.94 ± 17.38). These differences suggest that participants in each condition followed the respective instructions.

### Spectral EEG

Phase 2: In order to evaluate differences between groups on EEG tonic power changes across the frequency bands, a series of 2 (Condition: AC and CC) × 3 (ROI) mixed ANOVA’s were conducted on tonic change indices for each frequency band (Alpha, Beta, and Theta) during EO and EC – where condition was a between-subjects factor, and ROI was a within-subjects factor.

**FIGURE 1:**
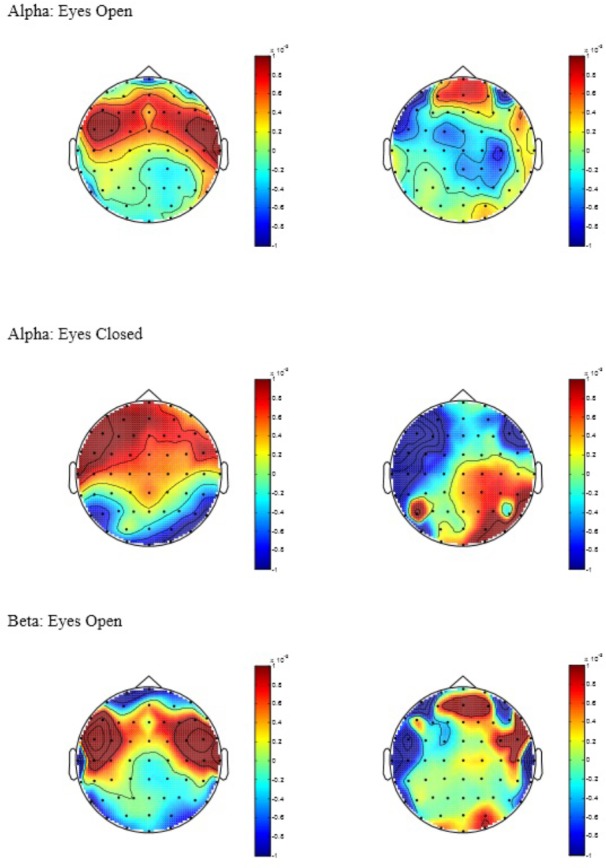
Topographic plots of tonic power change in Alpha and Beta, with AC to the left of the figure and CC to the right. Power values are expressed in log units of 10^∗^log_10_(μV^2^/Hz).

Alpha: a significant main effect of condition was observed [*F*(1,34) = 4.25, *p* = 0.04] indicating elevated change in global Alpha activity for the AC in comparison to CC during EO. Despite a insignificant interaction (*p* = 0.37), inspection of the between-group comparisons confirmed that this effect was most evident in the Midline ROI [*F*(1,34) = 4.66, *p* = 0.04, *d* = 0.80]. In addition, a significant condition by ROI interaction effect was observed [*F*(2,68) = 4.02, *p* = 0.02] for Alpha during EC. Univariate analysis confirmed that this was caused by a significant group difference in the Anterior ROI [*F*(1,34) = 4.74, *p* = 0.04, *d* = 0.76] indicating elevated change in Alpha activity for AC in comparison to CC. No differences were observed for Midline or Posterior ROIs (*p*’s > 0.05) during both EO and EC. Beta: no significant main or interaction effects were observed for Beta-band activity during EC (*p*’s > 0.05). However, a significant main effect of condition was observed [*F*(1,34) = 4.91, *p* = 0.034] indicating elevated change in global Beta activity for AC in comparison to CC during EO. Theta: no significant main or interaction effects were observed for Theta-band activity during EO or EC (*p*’s > 0.05) – **Figure 1** displays topographic plots representing the significant between-group differences in tonic change for Alpha during EO and EC and Beta during EO. To help supplement further interpretation of the overall between group differences, the means, standard deviations, and between-group Cohen’s *d* effect sizes and 95% confidence intervals (CI) were calculated (see **Table 1**). Inspection of the means indicated that in general, the AC yielded a positive (increase) change in spectral band power across a majority of the ROIs for both the EO and EC conditions. In contrast, the CC appeared to yield a negative (decrease) change in spectral band power across a majority of ROIs for EO and almost half the ROIs for EC. These data thus indicate that the direction and pattern of change largely differed according to group: for the AC, greater positive change was observed to occur in the Anterior, followed by the Midline, followed by the Posterior ROIs in both Alpha and Beta across EO and EC. Theta, on the other hand, demonstrated minimal change across ROIs for both EO and EC. In contrast, the CC showed less consistency between ROIs in Alpha and Beta during EO and EC, and demonstrated greater negative ROI change in Theta-band activity across EO and EC (with the Anterior ROI most pronounced).

**Table 1:** Means and standard deviations of tonic power change across eyes-open and eyes-closed ROIs.

			Active Condition (*n* = 18)		Control Condition (*n* = 18)			
Condition	Band	ROI	Mean	SD	Mean	SD	Cohen’s *d*	95% CI
**Eyes Open**								
	Alpha	Anterior	0.0008	0.0019	−0.0001	0.0019	0.487	0.467, 0.488
		Midline	0.0005	0.0009	−0.0002	0.0009	0.800^∗^	0.800, 0.801
		Posterior	0.0000	0.0005	−0.0001	0.0010	0.130	0.130, 0,131
	Beta	Anterior	0.0013	0.0005	0.0001	0.0018	0.567	0.566, 0.568
		Midline	0.0005	0.0026	−0.0010	0.0032	0.529	0.528, 0.530
		Posterior	−0.0002	0.0009	−0.0003	0.0009	0.114	0.114, 0.115
	Theta	Anterior	0.0000	0.0026	−0.0009	0.0030	0.330	0.329, 0.331
		Midline	−0.0003	0.0014	−0.0004	0.0019	0.062	0.061, 0.062
		Posterior	−0.0002	0.0007	−0.0004	0.0010	0.238	0.238, 0.239
**Eyes Closed**								
	Alpha	Anterior	0.0009	0.0033	−0.0019	0.0042	0.763^∗^	0.762, 0.764
		Midline	0.0005	0.0014	−0.0004	0.0025	0.457	0.456, 0.458
		Posterior	−0.0003	0.0015	0.0004	0.0019	−0.421	−0.420, −0.421
	Beta	Anterior	0.0004	0.0015	−0.0011	0.0043	0.479	0.478, 0.480
		Midline	0.0003	0.0015	0.0002	0.0028	0.229	0.228, 0.230
		Posterior	−0.0001	0.0004	0.0002	0.0020	−0.214	−0.214, −0.215
	Theta	Anterior	0.0002	0.0012	−0.0014	0.0050	0.453	0.452, 0.454
		Midline	−0.0001	0.0011	0.0003	0.0026	−0.206	−0.206, −0.207
		Posterior	0.0000	0.0008	0.0003	0.0018	−0.222	−0.221, −0.222

*^∗^P < 0.05. Negative values indicate a decrease in power, and positive values indicate an increase in power. Between-group Cohen’s d effect sizes and 95% CIs are presented to the right of mean values and standard deviations. ROI = Region of Interest.*

Phase 3: In order to evaluate associations of band-power change within and across ROIs, a series of exploratory bivariate correlational analysis were conducted across EO and EC for both conditions. Positive frontoparietal associations were observed between Alpha and Beta during EO, but these were only found to be significant in the AC (*r*’s = 0.83 and 0.50, for Anterior and Midline respectively). In addition, the AC yielded significant positive frontoparietal associations between Alpha and Beta during EC (*r*’s = 0.58 and 0.77, for Anterior and Midline respectively), which were only observed in the Anterior ROI for the CC (*r* = 0.84). These data thus indicate that the frontoparietal changes in Alpha were generally accompanied by changes in Beta in the same ROI. However, these associations were largely significant in the AC rather than the CC. In addition, inspection of within group correlations between ROIs for both Alpha and Beta were investigated to determine level of oscillatory synchrony between frontoparietal areas. As suspected, significant positive associations were observed between Anterior and Midline ROIs for Alpha during EO and EC in the AC (*r* = 0.68 and 0.66, respectively) but not the CC (*r*’s = 0.16 and 0.31, respectively). Similarly, significant positive associations were also observed between Anterior and Midline ROIs for Beta during EO and EC in the AC (*r*’s = 0.7 and 0.8, respectively) but not the CC (*r*’s = 0.02 and −0.25, respectively). These data indicate that frontoparietal changes in Alpha and Beta were highly correlated between Anterior and Midline ROIs, however these associations were only significant in the AC rather than the CC. Finally, level of asymmetry between alpha ROIs was investigated to determine whether enhancement of Anterior regions led to suppression over Posterior sites. Both the AC and the CC demonstrated Alpha asymmetry (negative correlation), but this effect was again only significant in the AC (*r* = −0.92).

## Discussion

The present study is the first to demonstrate that a single dose of the Attention Training Technique enhances resting alpha and beta-oscillations in frontoparietal networks known to be implicated in top-down attention and executive control. As predicted, participants in the AC showed significant elevated change in frontoparietal alpha and beta-band activity. Furthermore, anterior and midline ROIs in both alpha and beta were significantly correlated in the AC indicting greater degrees of neuronal synchrony. In contrast, limited theta-band activity was observed in both the AC and CC. This oscillatory signature distinguishes ATT from other forms of treatment that employ attention modification tasks. For example, studies evaluating the effects of autogenic relaxation training and mindfulness-based techniques have shown increased theta-band activity in association with relaxed, drowsy states (e.g., Brown, 1974; Austin, 1999; Chan et al., 2011); a finding also commonly associated with various forms of meditation (e.g., Delmonte, 1984; Andresen, 2000; Travis and Shear, 2010). In addition, such studies also tend to report either little to no change in beta-band activity (Dunn et al., 1999; Cahn and Polich, 2006) and/or decreased frontoparietal beta-band activity (e.g., Ikemi, 1988; Jacobs et al., 1996).

The role of beta-band activity has received growing interest due to a wealth of animal and human studies indicating that beta-band enhancement reflects engagement of frontoparietal networks assumed to be involved in top-down attentional control (e.g., Bisley and Goldberg, 2003; Gross et al., 2004; Basile et al., 2007; Swann et al., 2009). In addition, alpha and beta-band enhancements are observed to co-occur during tasks involving information retrieval and selective attention (Zanto and Gazzaley, 2009), and both are reported to strongly correlate in recent biologically plausible neural network models evaluating working memory abilities (Lundqvist et al., 2011). These findings have given rise to the hypothesis that both frequencies may serve similar neurocognitive functions (Waldhauser et al., 2012). Given that ATT is designed to improve top-down attentional control and flexibility over competing sources of information, the observed combination of enhanced alpha, and beta sits in agreement with these findings. From a conceptual point of view, these findings also provide support for the hypothesis that ATT’s neuronal mechanism of change may lie in the training of frontoparietal areas associated with top-down executive control. Indeed, recent imaging studies evaluating ATT have also reported similar findings; Rosenbaum et al. (2018) interpreted their results as evidence of ATT increasing areas of the cognitive control network and dorsal attention network (they also go on to point out that aberrant functioning in both these areas are known to lead to negative emotional outcomes).

In addition to identifying ATT’s oscillatory profile, the current findings also highlight the important implication of engaging with the ATT instructions. As predicted by S-REF theory, those who passively experienced ATT without engaging in the technique (CC) showed static or decreased change in anterior and midline ROIs for both alpha and beta. This may suggest that it is not exposure to ATT *per se* which yields neurocognitive change, but the degree to which individuals engage in the attentional tasks. This finding was further supported by significant alpha asymmetry observed in the AC in contrast to the CC. Evidence suggests that alpha enhancement of frontoparietal networks associated with sustained and directed attention correlates negatively with posterior amplitude (e.g., Corbetta and Shulman, 2002; Jensen and Mazaheri, 2010). Greater alpha asymmetry in the AC is therefore interpreted as reflecting greater levels of engagement in the attentional tasks. Furthermore, the presence of significant alpha asymmetry again separates ATT from other forms of attention modification, such as mindfulness and meditation, that tend to show aligned anterior-posterior alpha symmetry (e.g., Satyanarayana et al., 1992; Lagopoulos et al., 2009) and/or midline-posterior asymmetry (e.g., Ivanovski and Malhi, 2007; Chiesa and Serretti, 2010).

These findings also have an important clinical implication when considered in the context of reduced prefrontal functioning, which has been widely observed across multiple psychiatric conditions (e.g., MacDonald and Carter, 2003; Blumberg et al., 2004; Meyer et al., 2004; MacDonald et al., 2005). For example, prefrontal dysfunction characterized by diminished tonic alpha power has been reliably observed in schizophrenic patients (e.g., Sponheim et al., 1994, 2000) and in studies investigating the neurophysiology of depression and anxiety (e.g., Henriques and Davidson, 1990; Thibodeau et al., 2006). However, common psychological treatments such as cognitive remediation (for reviews, see Kurtz, 2003; Bellack, 2004) and computerized attention modification paradigms (e.g., Amir et al., 2009; Bar-Haim, 2010) regularly struggle to yield superiority above treatment as usual and often fail to explicitly link change in neurophysiology with the techniques being applied (Siegle et al., 2007). In contrast, ATT is a clinically reliable strategy aimed at enhancing global top-down attentional and executive control which has now been shown to enhance tonic alpha and beta power in frontopareital networks. Although the current results were not directly evaluated in association with clinical phenomena, it seems reasonable to assume that the neurophysiological effects of ATT may be implicated in the improvement of prefrontal functioning.

This study has some important limitations. First, as noted above, these results are unable to determine whether the observed neurophysiological changes are accompanied by symptom reductions in clinical populations. Assessing this prospect will involve repeated measurement of tonic alpha and beta-band change during a full course of ATT treatment with a clinical sample in comparison to a control. This will also help determine whether ATT yields a dose-response effect in parallel with increased symptom change. Second, although this study was able to control for trait measures of attentional control and flexibility, and a state measure of current mood, we did not employ an attention-related behavioral measure. Furthermore, despite efforts to ensure successful randomisation and counterbalancing, this study was unblinded to the experimenter. Thus, future replications will benefit from blinded replications with supplemented measures of top-down attentional control. Third, given the small sample size, we are unable to determine whether some of the negative findings are false negatives; the trends toward significance here may reach significance with larger sample sizes.

## Conclusion

To our knowledge this is the first EEG study to evaluate the neurophysiological effects of ATT. A single dose of the treatment was observed to yield significant tonic alpha and beta-band enhancement in frontoparietal networks known to be implicated in top-down attentional and executive control. The specific effect of enhanced frontoparietal alpha and beta-band activity in combination with static theta-band activity suggests ATT yields a different oscillatory signature to other forms of intervention such as mindfulness and meditation-based strategies. There is growing clinical and analog evidence to suggest that ATT exerts strong therapeutic effects. These preliminary data suggest that the biological effects of ATT can be readily detected, may be equally promising and present an exciting opportunity for new lines of enquiry examining its neural substrates.

## Author Contributions

AW conceived the study idea and supervised the study. MK and AW designed the study and analyzed the data. MK collected the data. Both authors contributed to writing the manuscript.

## Conflict of Interest Statement

The authors declare that the research was conducted in the absence of any commercial or financial relationships that could be construed as a potential conflict of interest.

## Footnotes

1 http://www.randomizer.org/

2 http://www.biosemi.com/faq/cms&drl.htm
